# Electrified externally heated rotary calciner for calcination of cement raw meal

**DOI:** 10.1016/j.heliyon.2023.e22023

**Published:** 2023-11-04

**Authors:** Ron M. Jacob, Jean-Patrick Pinheiro, Lars-André Tokheim

**Affiliations:** a University of South-Eastern Norway, Kjølnes ring 56, 3918, Porsgrunn, Norway; b Institute for Energy Technology, Instituttveien 18, 2007, Kjeller, Norway

**Keywords:** Electrified calciner, Rotary kiln, CO_2_ emissions, Scale-up

## Abstract

The cement industry can reduce its CO_2_ emissions by electrifying the calciner. It can avoid emissions from fuel combustion and produce pure CO_2_ from the calcination reaction (CaCO_3_ → CaO + CO_2_) for direct capture. A differential-algebraic equation (DAE) model of an electrified rotary calciner was developed and validated against experimental results. The heat transfer coefficient was around 30 W/(m^2^K), with the calciner inclined at 15°. This value increased to 80 W/(m^2^K) by reducing the inclination to 2°. The rotary calciner for producing 1 Mton/yr clinker with an internal diameter of 5 m needs a length of 485 m to reach a calcination degree of 94 %. The large system size suggests that this calciner may not be suitable for full-scale production. However, it can still be used for small-scale green production of calcined limestone.

## 1 Introduction

The cement industry has the second largest contribution to industrial CO_2_ emissions, amounting to 2.52 Gt in 2021 [1]. Around two-thirds of these emissions come from the decomposition of limestone (the main ingredient in cement raw meal) [2,3], which occurs through the calcination reaction (CaCO_3_ → CaO + CO_2_). Typically, more than 90 % of the calcination happens inside the calciner, which is directly heated by fuel combustion [4,5]. The exhaust from the production process contains different gases such as CO_2_, N_2_, O_2_, NO_x_, SO_x_, and CO_2_ capture technologies are required to separate CO_2_ from this mixture.

Several CO_2_ capture technologies, such as post-combustion, oxy-fuel combustion, and direct capture technologies, can be used in the cement industry [6]. Implementing CO_2_ capture technology will affect the capital costs due to the extra equipment required, and the operating costs due to an increased energy demand to capture the CO_2_. The CO_2_ is captured after the combustion in the post-combustion technology. Amine scrubbing [7] and calcium looping [8] are examples of this technology. Amine scrubbing utilizes an amine-based solvent, while calcium looping uses a calcium-based sorbent (mainly limestone) to selectively react with CO_2_ from the flue gas. The CO_2_ bound to the solvent or the sorbent can then be removed and captured in a separate reactor (a desorber in the scrubbing process or a calciner in the calcium looping process). The biggest challenge with post-combustion technology comes from the high energy required to release the absorbed CO_2_ [6]. Oxy-fuel combustion utilizes pure O_2_ instead of air for fuel combustion to achieve a high concentration of CO_2_ in the flue gas for direct capture [9]. Oxy-fuel technology has an energy penalty from the air separation unit (ASU), which separates oxygen from air. Furthermore, most of the equipment in clinker production may be affected, which can significantly affect the capital costs [6]. Direct capture technology directly captures the CO_2_ produced from limestone decomposition inside the calciner by heating it from the outside. Such a technology is currently being investigated at pilot-scale in the LEILAC (Low Emission Intensity Lime & Cement) project [10]. Even though direct capture handles the emissions from limestone decomposition, the emissions from fuel combustion must be captured with a separate method to further reduce the emissions.

The share of renewable energy for electricity generation is increasing and once a completely decarbonized electricity is available, the external heating in direct capture technology could be supplied through electrical energy. This technology was studied in a previous article [5] and the results indicated that such a system could reduce CO_2_ emission by 78 % compared to a coal-fired calciner system. The article also discussed the impact of calciner design. Calciner designs such as entrainment calciner require a very high gas recycling in the system as this gas is needed to entrain the particles in the system. The high gas recycling in the system puts approximately 21 % higher energy demand when compared to designs requiring no gas recycling. This excess demand comes mainly from an increased heat duty in the system to preheat the recycling gas. So, calciner designs requiring no gas recycling may offer lower energy penalties when compared to a high gas recycling system.

The drop tube calciner and the rotary calciner are designs that need no gas recycling. In the former, the cement raw meal is dropped inside an externally heated vertical tube. The external heat to the tube provides energy for meal preheating and calcination, and the particles move due to gravity. As mentioned above, the drop tube design is currently being investigated in the LEILAC project [10]. The drop tube calciner can be operated with either a counter-current or co-current setup. In the counter-current operation, the gas outlet is located at the top of the tube, i.e., the meal inlet end, while in the co-current operation, the gas outlet is located at the bottom of the tube, i.e., the meal outlet end. Operating with a counter-current design has some uncertainties related to meal entrainment from potentially high gas velocities. One could mitigate the problem by expanding the tube, but this can affect the heat transfer in the system. The co-current design also has some uncertainties as the meal may form clusters, and the residence time of the meal may be reduced [11]. Regardless of the challenges, the first phase of the LEILAC project showed promising results, so LEILAC 2 was established at a four times larger scale than the first phase [12]. LEILAC 2 will test multiple heat sources, including electrical heating on the external wall. However, this is ongoing research work, and the results are not yet publicly available.

A rotary calciner is another design alternative that requires no gas recycling. In such a design, the cement raw meal travels through an inclined cylinder rotating at a certain speed. The rotating drum improves radial mixing inside the bed, and particles travel mainly under gravity in the slightly inclined drum. The electrified externally heated rotary calciner is commercially available by some manufacturers [[13], [14], [15], [16]]. Recently, a research institute in Finland (VTT) also announced successful trial tests with this design at pilot-scale with a particle feeding of around 100 kg/h [17]. This means that the technology readiness level (TRL) of the design is relatively high, and the cement manufacturers could potentially replace a fuel-fired calciner with an electrified version.

Cement raw meal consists of cohesive particles which tend to move as a plug inside a rotating drum. This movement tends to reduce the heat transfer in the raw meal [18,19]. An externally heated rotary kiln has been studied in the literature [[20], [21], [22]]. However, to the best of author's knowledge, the literature does not cover the overall heat transfer coefficient in the rotary calciner for calcining raw meal.

This work aims to determine the overall heat transfer coefficient in an electrified externally heated rotary calciner. To reach the main aim, the objectives of this study are to i) establish a heat transfer and calcination kinetics model in an electrified externally heated rotary calciner, validated against lab-scale experimental results, and ii) determine the overall heat transfer coefficient. A differential-algebraic equation (DAE) model of the rotary calciner is developed in this study, and OpenModelica v1.19.2 is utilized for simulations of the DAE model. The model is then used to determine the overall heat transfer coefficient, which is required to scale up the design. Finally, the results are utilized to dimension the tube of an electrified rotary calciner when the raw meal feeding rate is 220 t/h, corresponding to a clinker production of about 1 Mt/y.

## 2 Experimental method

### 2.1 Experimental setup

**Fig. 1:**
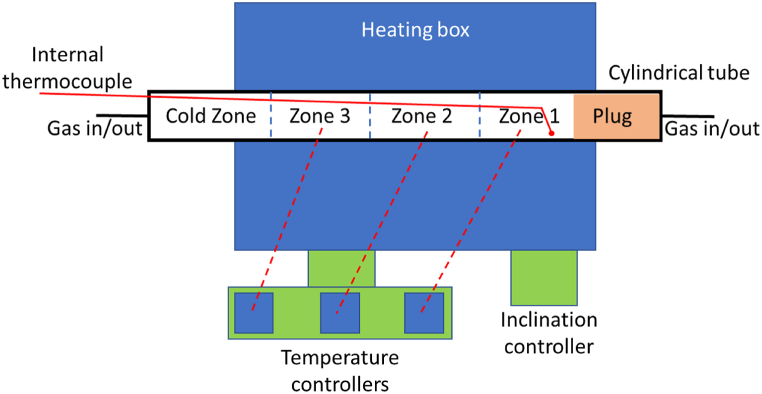
Experimental setup.

The experimental setup is shown in Fig. 1. The experimental setup consists of 1) Cylindrical tube made from Ni–Cr alloy (Sandvik 7RE10), 2) Heating box with Silicon Carbide elements, 3) Temperature controller, 4) Inclination controller, 5) Internal thermocouple, 6) Insulating plug at one end of the tube, and 5) Gas inlet/outlet section at both tube ends.

The cylindrical tube has an inner diameter of 68.8 mm and a thickness of 3.65 mm. The tube can be rotated at 37 RPM and can be inclined in the range 0–15° with the help of an inclination controller. The cylindrical tube has four zones. The first zone is the cold zone, which was exposed to the environment and used for raw meal feeding. The second and third zones are Zone 2 and Zone 3, which were fixed at 650 °C with controllers (this temperature was set in all experiments). Finally, the fourth zone is Zone 1, which was fixed at 975, 1000, or 1025 °C, depending on the experimental run. The total length of the hot zone is 1165 mm, wherein the length of zone 1 and 3 is 420 mm each, while zone 2 length is 325 mm.

The zone 1 end was permanently sealed with an insulating plug, while the cold end can be opened and closed during the experiments. The calciner tube can be flushed with either N_2_ or CO_2_, and the gas can flow in both directions. This was done by providing a gas inlet/outlet at both ends of the calciner tube. The gas flow rate was adjusted with a rotameter.

During the experiments, the internal thermocouple was immersed inside the meal bed. So, the temperature of the meal was measured with this thermocouple. The tip of the internal thermocouple was fixed at 6 cm from the plug. The voltage drop and current across each zone were also continuously measured, and these measurements were used to calculate the power across each zone based on Ohm's law.

### 2.2 Raw meal characteristics

**Fig. 2:**
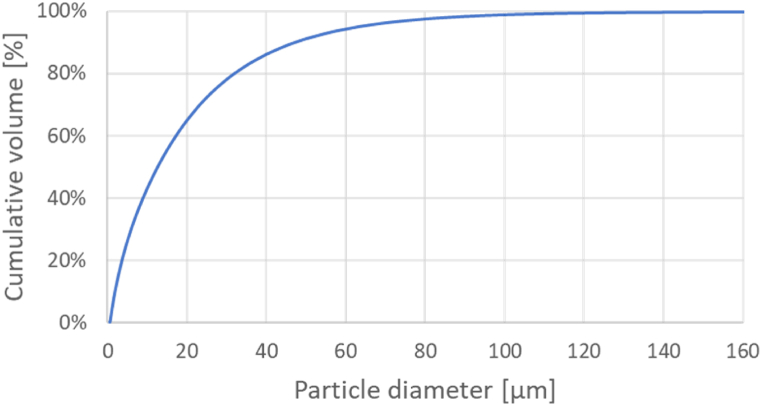
Particle size distribution of cement raw meal.

The particle size distribution fo raw meal is shown in Fig. 2. The particles had a weighted average of 21 μm, and a Sauter mean diameter of 5 μm. The bulk density of the material was measured and was found to be 1053 kg/m³.

The raw meal composition was also measured with XRF analysis before the experiments. The loss on ignition of the raw meal was 33.2 wt%, and the results from the XRF analysis are shown in Table 1. The raw meal composition is back-calculated for modelling purposes (also shown in Table 1). The sulphur trioxide (SO_3_) is assumed to exist as either sodium sulphate (Na_2_SO_4_) or potassium sulphate (K_2_SO_4_). The left sulphur trioxide (due to insufficient K_2_O and Na_2_O) is assumed to exist as calcium sulphate (CaSO_4_). The remaining lime (CaO) after CaSO_4_ is then assumed to exist as calcium carbonate (CaCO_3_).

**Table 1:** Raw meal composition (wt%). The loss on ignition (weight loss) is 33.2 %.

Component	XRF analysis, LoI-free basis (wt%)	Back-calculated raw composition (wt%)
CaCO_3_	–	77.2
CaO	44.05	–
SiO_2_	13.68	13.5
Al_2_O_3_	3.26	3.2
Fe_2_O_3_	1.96	1.9
MgO	1.79	1.8
K_2_O	0.86	–
Na_2_O	0.25	–
SO_3_	1.25	–
Na_2_SO_4_	–	0.5
K_2_SO_4_	–	1.6
CaSO_4_	–	0.3

### 2.3 Experimental procedure

200 gm of cement raw meal were inserted in the cold zone, and cold zone end was closed. The temperature set-point in each heating zone was set as discussed in the experimental setup section and the heating zones started to heat up. The tube was flushed with nitrogen from the cold zone end at a flow rate of 400 Nml/min to keep the raw meal cool and to remove all the air inside the drum during the heat up phase. After the heat-up of zones, nitrogen was replaced with carbon dioxide. Carbon dioxide was flushed for 2 h to create a pure CO_2_ environment. The direction of CO_2_ flushing was then changed from the cold zone end to the zone 1 end, and the flow rate was reduced to 150 Nml/min. This was done to minimize losses by convection during the tests. The tube was tilted by 15°, and rotation was started at 37 RPM. At this stage, the raw meal travelled to the zone 1 end. The measurements of power and temperature are reported from this stage. After 15 min, the tube was tilted to 0°, and the cold zone end was opened. The tube was then tilted by 15° in the opposite direction so that the meal travelled back from zone 1 and exited from the cold zone end. The meal was collected, and all the agglomerates were manually broken.

The calcination degree is then measured on the collected particles. 10 g of collected particles are further calcined in a muffle furnace at 950 °C for 5 h in two batches. The weight loss of the particles is measured, and the degree of calcination ($X_{c}$) is calculated by equation (1).

(1) $$X_{c}=\frac{100\times m_{Final}-\left(100-LOI\right)\times m_{Initial}}{m_{Final}}\times \frac{100}{LOI}$$

Here, $m_{Final}$ is the final sample mass, $m_{Initial}$ is the initial sample mass, and LOI is the loss on ignition for the raw meal.

## 3 Modelling method

The governing equation of the differential algebraic equation (DAE) model is described in this section. The DAE model was implemented in OpenModelica v1.19.2. OpenModelica is an open-source modelling tool for solving DAE equations and further information may be found in the user manual [23]. The model was discretized into 500 points for simulations.

### 3.1 Molar balance and reaction kinetics

The primary reaction of raw meal is the calcination reaction, which converts calcite (CaCO_3_) into lime (CaO) by the reaction CaCO_3_ → CaO + CO_2_. The lime can react with silicate to produce belite [24], which is a slow process controlled by diffusion mechanisms [25]. So, the belite formation is assumed to be negligible in this work.

The change in moles of each species j of raw meal ($n_{j}$) is given by equation (2) by assuming that the molar change due to chemical reaction is uniform in all directions. $\vartheta_{j}$ is the stoichiometric coefficient based on the calcination reaction (i.e. $\vartheta_{CaCO3}$ = −1, $\vartheta_{CaO}$ = 1, and $\vartheta_{CO2}$ = 1. For all other species, such as SiO_2_, Al_2_O_3_, etc., this coefficient is 0). $A_{c}$ is the surface area of the calcite, and $r_{C}$ is the calcination reaction rate [mol/(m^2^·sec)].

(2) $$\frac{{dn}_{j}}{dt}=\vartheta_{j}r_{C}A_{c}$$

The calcination kinetics is assumed to follow a shrinking core model, wherein the layer of calcite core shrinks as the reaction proceeds. The reaction rate is determined by interrelationships between three processes, i.e., 1) heat transfer to the calcite shell, 2) calcite decomposition, and 3) diffusion of CO_2_ through the porous product (CaO) layer [26]. The diffusion of CO_2_ and heat transfer through the porous product may become a major resistance for large particles. However, for particles in the micrometer range, the effect is likely very low [27]. So, heat transfer to the particle surface and calcite decomposition is assumed to control the reaction kinetics. The heat transfer mechanism is further discussed in the next section, whereas this section covers calcite decomposition.

The calcite decomposition starts when the equilibrium pressure ($p_{eq}$) [Pa] for the reaction is larger than the partial pressure of CO_2_ at the calcite surface ($p_{co2}$) [Pa], and the equilibrium pressure is given by equation (3). The kinetics of reaction ($k_{D}$) [mol/(m^2^sPa)] is given by equation (4), and the rate of reaction ($r_{c}$) is given by equation (5) [28].

(3) $$p_{eq}=4.192\times 10^{12}\;\exp\left(\frac{-20474}{T_{m}}\right)$$

(4) $$k_{D}=1.22\times 10^{-5}\;\exp\left(\frac{-4026}{T_{m}}\right)$$

(5) $$r_{C}=k_{D}\left(p_{eq}-p_{co2}\right)A_{eff}$$

The pore-to-particle area ratio ($A_{eff}$) is added in equation (5) to account for the excess reaction area coming from the porous CaCO_3_ particles. The ratio is in the range 1–5 depending on the type of limestone in the raw meal [29].

**Fig. 3:**
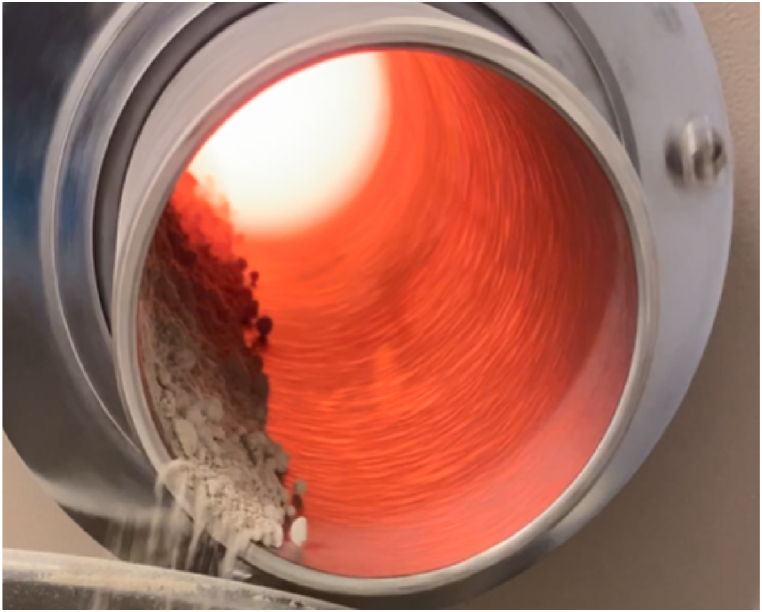
Outflow of agglomerated particles from the rotary calciner.

The calcite core is assumed to be spherical, and the initial surface area is calculated based on the initial diameter of the particle. The average diameter of particles is 21 μm; however, the particle tends to form agglomerates inside the calciner. In a previous calcination study [26], calcite particles of 10–15 μm were reported to agglomerate into a sphere of around 1 cm. The agglomerated particles had the highest conversion at the surface and the lowest at the center, which complies with the shrinking core model. As shown in Fig. 3, the particles in the rotary calciner were visually observed to form agglomerates of around 1 mm, so this value was fixed as the initial diameter of the calcite core.

### 3.2 Energy balance and heat transfer

The enthalpy of the raw meal ($H_{m}$) changes during the heat transfer process and this is given by equation (6). Here ${\dot{q}}_{net,m}$ is the net heat transfer from the heater to the raw meal during heat-up and calcination.

(6) $$\frac{{dH}_{m}}{dt}={\dot{q}}_{net,m}$$

The total enthalpy of the raw meal is the summation of the product of moles of each species j and their specific enthalpy ($H_{j}$, documented in Appendix A), which is given by equation (7). The total enthalpy increases when the transferred heat (or ${\dot{q}}_{net,m}$) affects the sensible heat (wherein $H_{j}$ changes with temperature based on correlations in Appendix A), or heat of reaction (wherein $n_{j}$ changes based on equation (2) and $H_{j}$ changes based on the correlations in Appendix A).

(7) $$H_{m}=\sum n_{j}H_{j}$$

As discussed in the previous section, heat transfer is an important aspect that controls the calcination mechanism. The heat is transferred from the inner wall of the tube to the raw meal through 1) conduction/convection from the wall surface in direct contact with the meal, 2) radiation from the exposed wall surface to the meal surface, and 3) convection from the exposed wall surface to the gas and then from the gas to the meal. The gas flow rate is kept relatively low during the experiments, so the convection heat transfer from the gas flow (third point) is assumed to be negligible.

The heat transfer from the wall in direct contact with the meal (${\dot{q}}_{cw}$) is given by equation (8). Here, $A_{cw}$ is the surface area of the contact wall, and $T_{w,in}$ is the temperature of inner wall of the tube.

(8) $${\dot{q}}_{cw}=h_{cw,m}A_{cw}\left(T_{w,in}-T_{m}\right)$$

The heat transfer coefficient from the contact wall to the meal ($h_{cw,m}$) can be calculated with empirical models documented in the literature [30,31]. However, all the correlations are developed for rolling motion where the bed is well-mixed. The raw meal particles are cohesive and display a sliding motion inside the calciner. Due to this phenomenon, the bed is not well mixed, and all the empirical models tend to over-predict the heat transfer coefficient by a factor of 2 [18] to 5 [19]. So, instead of using an empirical model, the heat transfer coefficient is assumed to be 40 W/(m^2^K), which is an approximate value from a previous study [18].

**Fig. 4:**
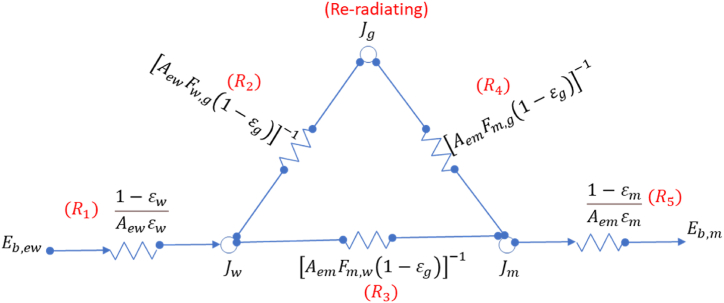
Radiation network to determine heat transfer.

The radiation heat transfer from the exposed inner wall to the meal surface is very complex as there are interactions between many participating media, i.e., meal, wall, and gas. The radiation heat transfer can be calculated with a network analysis [32,33], and the network is shown in Fig. 4. $R_{1}\ldots R_{5}$ are the network resistances [1/m^2^], $A_{em}$ is the area of exposed meal [m^2^], $A_{ew}$ is the area of exposed inner wall [m^2^], $J_{m}$, $J_{\text{ew}}$ and $J_{g}$ are the radiosities from meal, exposed wall, and gas, respectively [W/m^2^], and $E_{b,m}$, $E_{b,\text{ew}}$, $E_{b,g}$ are the black body emissive power fluxes from the meal, exposed wall, and gas, respectively [W/m^2^]. The CO_2_ gas is assumed to be re-radiating, i.e., it re-radiates all the incident heat.

The radiation heat transfer from the exposed wall to the exposed meal (${\dot{q}}_{\text{ew}}$) [W] is then given by equation (9).

(9) $${\dot{q}}_{ew}=\frac{\sigma }{R_{\text{eff}}}\left({T_{w,in}}^{4}-{T_{m,s}}^{4}\right)$$

Here, $R_{\text{eff}}$ is the effective resistance in the network [1/m^2^], $T_{m,s}$ is the meal temperature at the bed surface [K]. The effective heat transfer resistance ($R_{\text{eff}}$) from the network is calculated from equation (10).

(10) $$R_{\text{eff}}=R_{1}+\frac{1}{\frac{1}{R_{3}}+\frac{1}{R_{2}+R_{4}}}+R_{5}$$

The emissivity of raw meal ($\varepsilon_{m}$) is assumed to have an emissivity close to limestone as it the main constituent ($\varepsilon_{m}$ can lie between 0.732 at 711 K and 0.676 at 1228 K [34]. Assumed to be 0.69). The wall is assumed have an emissivity ($\varepsilon_{w}$) of 0.88 based on reported value for Nickel Chromium alloy [35]. The calculation of other unknowns, such as emissivity of gas ($\varepsilon_{g}$), view factors ($F_{w,g}$, $F_{m,g}$, $F_{m,w}$), and exposed area ($A_{ew}$, $A_{em}$) is summarized in Appendix B.

The heat is first transferred to the meal surface. The heat must then travel inside the meal via convection (as the particles are constantly mixed and behave almost like a fluid) to reach the calcination temperature. The highest heat transfer resistance in convection lies in the boundary layer, which can be approximated with a linear temperature gradient within the conduction thickness (δ) [36]. The conduction thickness is smaller than the actual boundary layer thickness, which will depend on the operational conditions of the calciner, such as the mass of particles, rotational speed, and calciner inclination. According to this theory, the heat transferred from the exposed wall to the exposed meal (${\dot{q}}_{ew}$) has to meet the constraint given by equation (11).

(11) $${\dot{q}}_{ew}=\frac{k_{m}A_{em}\left(T_{m,s}-T_{m}\right)}{\delta }$$

Here, $k_{m}$ is the effective conductivity of the meal [W/mK] (assumed to be 0.14 W/(m.K) based on previous measurement in literature [18]), $T_{m}$ is the meal core temperature [K], and δ is the conduction thickness [m]. No attempt is made in this study to derive the conduction thickness. Since the conduction thickness is between 0 and the maximum bed height ($h_{p,m}$, documented in Appendix B), the conduction thickness is found experimentally by choosing the appropriate fraction of meal bed height ($f_{m}$) and inserting it into equation (12).

(12) $$\delta =h_{p,m}∙f_{m}$$

The net heat transfer to the meal from the inner wall (${\dot{q}}_{net,m}$) is then given by equation (13).

(13) $${\dot{q}}_{net,m}={\dot{q}}_{ew}+{\dot{q}}_{cw}$$

### 3.3 Heat transfer coefficient

The heat transfer coefficient is calculated for two process stages: 1) meal preheating, and 2) meal calcination.

During the meal preheating, the heat transfer coefficient ($h_{PH}$) is given by equation (14).

(14) $$h_{PH}=\frac{{\dot{q}}_{net,m,\text{PH}}}{A_{w,in}∙{\Delta T}_{LMTD}}$$

Here, ${\dot{q}}_{net,m,\text{PH}}$ is the net power transfer during the preheating phase, $A_{w,in}$ is the total area of inner wall, ${\Delta T}_{LMTD}$ is the log mean temperature difference which is further given by equation (15).

(15) $${\Delta T}_{LMTD}=\frac{\left(T_{w,in}-T_{m,c}\right)-\left(T_{w,in}-T_{m,in}\right)}{\ln\left(\frac{T_{w,in}-T_{m,c}}{T_{w,in}-T_{m,in}}\right)}$$

Here, $T_{m,c}$ is the calcination temperature [K], and $T_{m,in}$ is the inlet meal temperature [K]. The heat transfer coefficient in calcination phase ($h_{C}$) is then given by equation (16). Here, ${\dot{q}}_{net,m,C}$ is the heat transferred during the calcination stage.

(16) $$h_{C}=\frac{{\dot{q}}_{net,m,C}}{A_{w,in}∙\left(T_{w,in}-T_{m,c}\right)}$$

### 3.4 Scaling up the system

The heat transfer coefficient is used when scaling up the system. The dimension of the rotating tube in a rotary calciner is estimated based on the heat transfer coefficient calculated with formulas described in the previous section.

The duty of the calciner at in the preheating (${\dot{q}}_{net,m,\text{PH}}$) and calcination (${\dot{q}}_{net,m,C}$) stages can be calculated by the mass and energy balance of the system for a given raw meal feeding rate. The total heat transfer area ($A_{HT}$) at this meal feeding rate is then calculated by rearranging equations (15), (16) and using the heat transfer coefficients calculated in this work. Finally, assuming an internal diameter ($d_{c}$), the length of the calciner ($l_{c}$) can be calculated by using equation (17).

(17) $$l_{c}=\frac{A_{HT}}{\pi ∙d_{c}}$$

The dimension of the calciner with a raw meal feeding of 220 t/h, corresponding to a clinker production rate of 1 Mt/y, is presented in the results section using the outlined method.

## 4 Results and discussions

### 4.1 Model calibration

**Fig. 5:**
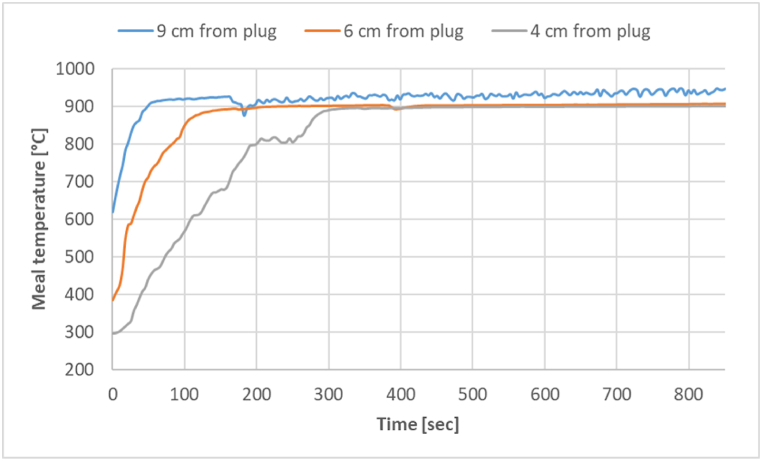
Measured meal temperature at three different thermocouple locations with zone 1 at 975 °C.

The experimental results of meal temperatures with thermocouples at three different locations are shown in Fig. 5.

The meal temperature first increases due to sensible heat and then reaches a plateau temperate where the reaction kinetics is counter-balanced by heat transfer. Over time, the reaction rate drops due to a reduced calcite surface area (shrinking core), which causes the temperature of the meal to rise slowly, which again increases the reaction rate. So, the plateau temperature is relatively constant at the beginning of the calcination phase, however, it should slowly rise due to the shift in the balance between heat transfer and kinetics. The thermocouple placed closest to the plug (4 cm from the plug) shows the lowest temperature, while the one located farthest (9 cm from the plug) shows the highest temperature. This effect comes from two factors: 1) When the thermocouple is closer to the plug, it is immersed at a higher bed depth. So, the radiated heat needs to diffuse a longer distance to reach the thermocouple location, giving the lower temperature. 2) The plug is not a perfect insulator, so there is some heat sink from this location. The temperature read by the thermocouple located 6 cm from the plug lies between the two extreme cases, so this temperature better represents the average meal temperature. Therefore, the temperature readings from the thermocouple at 6 cm from the plug are reported in all further results.

**Fig. 6:**
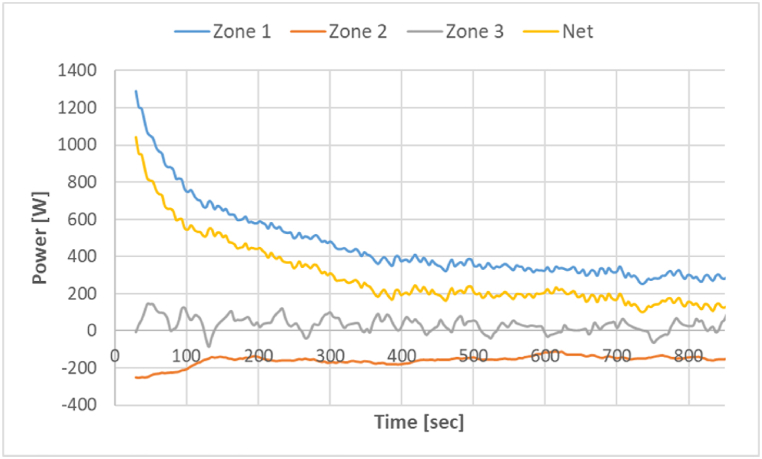
Measured power in each zone and the net power after raw meal entry with zone 1 at 975 °C.

The power transferred to the meal from each zone and the net power after raw meal entry to zone 1 is shown in Fig. 6 when zone 1 temperature is fixed at 975 °C. When the meal reaches zone 1 (at time = 0 s), the temperature difference between the meal and zone 1 is highest. So, the power transfer is highest in the beginning. After the onset of calcination, the power stabilizes as the raw meal temperature becomes almost constant. There is some heat sink from zone 1 to zone 2 due to lower temperatures in zone 2, so zone 2 produces a negative power (as it absorbs heat). The heat sink from zone 1 to zone 3 is almost negligible as it is far enough from the zone 1 end. The net power to the meal is given by adding power utilized in zone 1 and subtracting power absorbed in zone 2. All further results show only the net power transferred to the meal.

**Fig. 7:**
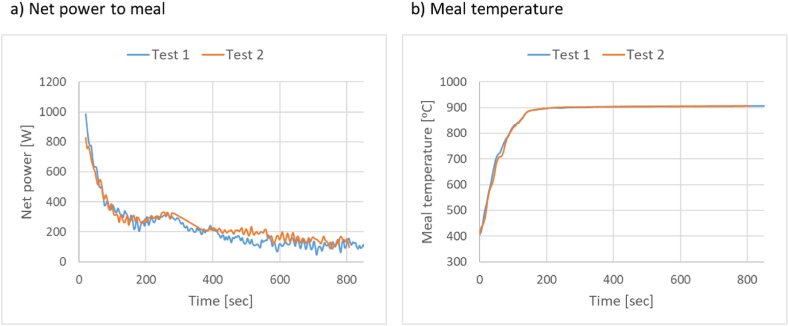
Repeatability tests of a) net power and b) meal temperature with zone 1 at 975 °C.

To measure the uncertainty of the measured power and temperature, a repetition of the experiments with zone 1 at 975 °C was conducted and the results are shown in Fig. 7(a and b). The results show a low deviation in the measured parameters with a repetition of the experiment.

**Fig. 8:**
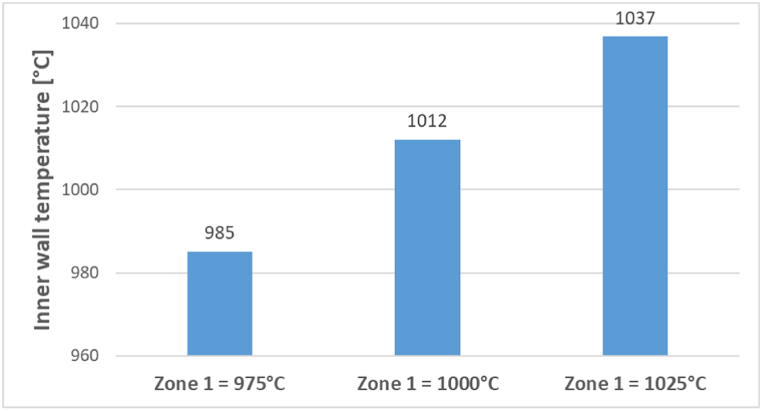
Measured inner wall temperature at three different zone 1 temperatures.

The inner wall temperature in zone 1 is measured before the meal entry at each zone 1 setpoint (i.e., 975, 1000, and 1025 °C) and is shown in Fig. 8. The measured inner wall temperature is higher than the zone 1 setpoint temperature as the zone 1 heating box captures the heat from all the walls, including the insulation wall. Since the insulation wall is exposed to the environment on the other side, the temperature is lower than the actual cylindrical tube temperature. The measured inner wall temperature is used in equations (8), (9) fixed as boundary conditions for the simulations.

**Fig. 9:**
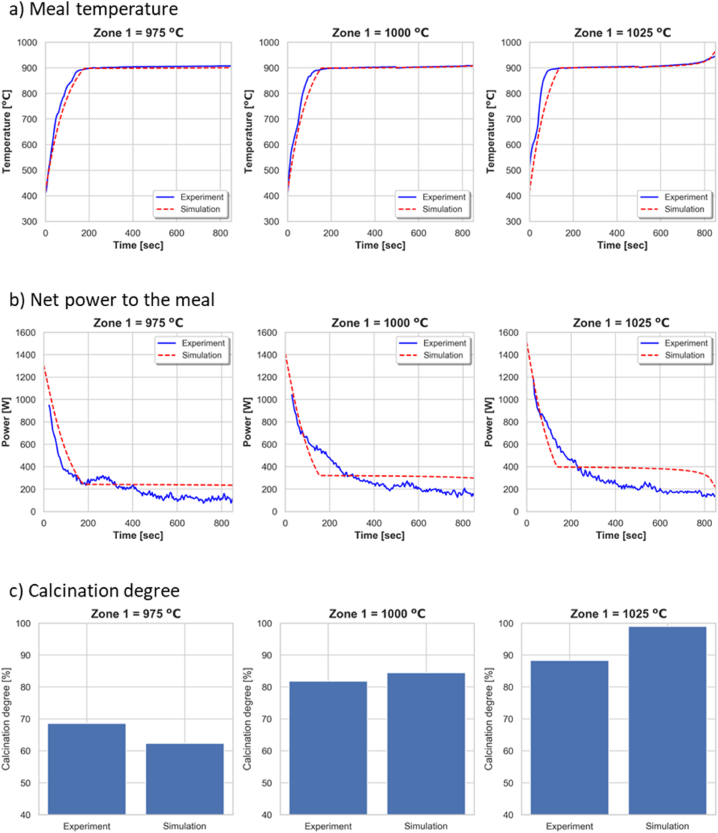
Comparison of a) meal temperature, b) net power to the meal, and c) calcination degree from experiments and simulations at three different zone 1 temperatures.

The meal temperature, net power, and calcination degree from the experiments and simulations at three different zone 1 temperature setpoints (i.e., 975, 1000, and 1025 °C) is shown in Fig. 9(a–c). The modelling parameters are: 1) fraction of meal bed height ($f_{m}$) = 0.6 %, 2) Pore-to-particle area ratio ($A_{eff}$) = 2. The modelling parameters are fixed for all the simulations. The simulation results comply quite well with the experimental results, so the model can be seen as successfully validated.

### 4.2 Heat transfer coefficient

**Fig. 10:**
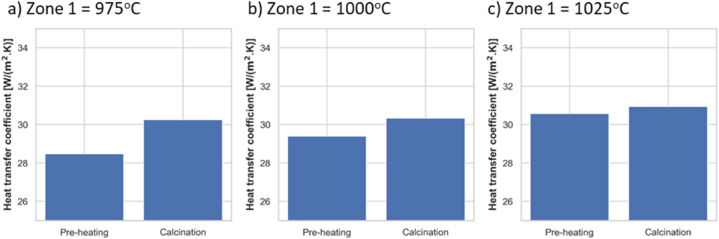
Comparison of heat transfer coefficient at a) Zone 1 = 975 °C, b) Zone 1 = 1000 °C, and c) Zone 1 = 1025 °C.

The heat transfer coefficient is calculated in different zone temperatures at the preheating and calcination stages, and the result is given in Fig. 10(a–c). The heat transfer coefficient increases with zone temperature as the effect of radiation become stronger. The heat transfer coefficient in the preheating stage is also higher than in the calcination stage due to the stronger radiation from higher meal temperature at this stage.

**Fig. 11:**
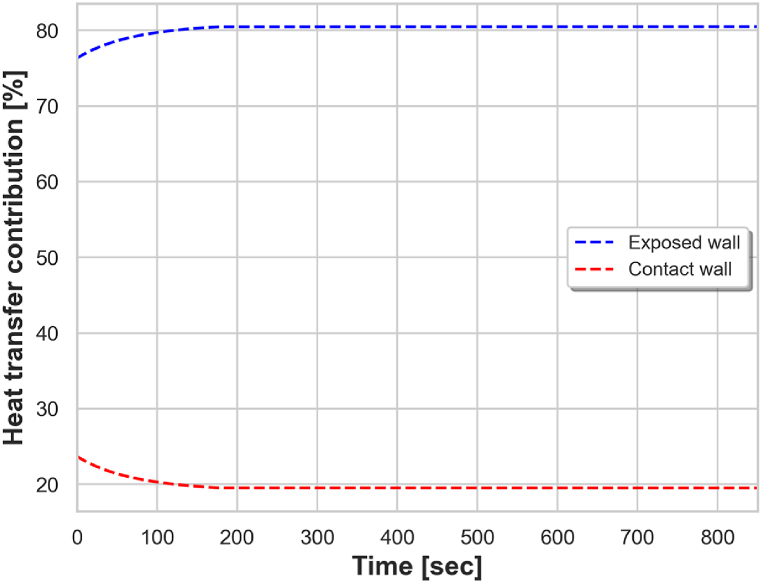
Contribution of heat transfer from exposed wall and contact wall with zone 1 at 975 °C.

The contribution of heat transfer from the contact surface and from the exposed surface for the case with zone 1 at 975 °C is shown in Fig. 11. The results clearly show that the heat transfer from the exposed surface is dominant in the studied temperature range, with the covered wall contributing only around 20 % of the heat transfer. This is due to the stronger effect of radiation at higher temperatures.

A continuously fed calciner typically operates at an inclination lower than 2°. A lower inclination degree provides a higher meal exposure area and, thereby, a higher heat transfer coefficient. So, the inclination of 15° during experiments under-estimated the actual heat transfer coefficient. The model was utilized to simulate the conditions with an inclination of 2°. The meal area, wall area, and view factor at an inclination of 2° used in the simulations are summarized in Appendix B. At an inner wall temperature of 1050 °C, the meal achieved a full calcination within 323 s. The heat transfer coefficient in the preheating and calcination stage becomes 78 and 81 W/(m^2^K), respectively. The results show that the heat transfer coefficient can be increased up to 2.5 times by reducing the inclination from 15° to 2°.

### 4.3 Scale-up results

The calculated heat transfer coefficients of 78 and 81 W/(m^2^K) in the preheating and calcination stages, respectively, were used for dimensioning the rotary calciner. A raw meal feeding of 220 t/h in a calciner with an internal diameter of 5 m needs a length of 485 m to achieve a calcination degree of 94 %. The length could be reduced by increasing the heat transfer coefficient, e.g. by placing internal lifters to improve meal mixing. This long rotary calciner will also have a high outer surface area, meaning that the heat loss from the outer surface will be large. If this long calciner is considered infeasible to build, an alternative could be to use four calciners, each with a length of 121 m. This should give approximately the same total heat transfer area. The construction of several smaller units means that the outer surface area becomes higher and thereby the heat loss increases further. The large size of the system suggests that the wall-heated rotary calciner is more suitable for small-scale production of calcined limestone.

## 5 Conclusions and future work

The heat transfer study of the externally heated rotary calciner shows that the main contribution to the heat transfer is from radiation, and the heat transfer coefficient increases with increasing wall temperature. The calciner inclined at 15° shows a low heat transfer coefficient of about 30 W/(m^2^K). An actual calciner should operate at an inclination lower than 2°, and the heat transfer coefficient increased to around 80 W/(m^2^K) for a calciner inclined at 2° due to an increased meal exposure area.

The dimensions of the rotary calciner to handle a feeding rate of 220 t/h were estimated based on the heat transfer coefficient. A calciner with an internal diameter of 5 m needs a length of around 485 m to achieve a calcination degree of 94 %. Alternatively, four calciners each with a length of 121 m may be used. The wall-heated rotary calciner may be a good option for small-scale green production of calcined limestone. However, scale-up of the concept to large-scale production is not recommended due to the large size of a full-scale system.

The study in this work is limited to a small-scale batch process. So, experimenting at a larger scale with continuous feeding is recommended in future work to improve the reliability of results from this work. The cement raw meal studied in this work is cohesive due to which it has poor particle mixing. Adding lifters or introducing coarse particles may improve the mixing behaviour, and this is recommended in future experiments. Another challenge comes from the design of the rotary calciner wherein a limited particle surface is exposed to the heating surface. If the particles are suspended inside the reactor, the exposure of the particle surface can increase, thereby increasing the heat transfer coefficient. However, suspending the particles would need other designs such as a drop tube calciner or a fluidized bed calciner. These designs are recommended to study in future work. Further, the model developed for the rotary calciner could also be utilized by simulating calcination of other materials, such as clay.

## Data availability statement

Data will be made available on request.

## CRediT authorship contribution statement

**Ron M. Jacob:** Writing – original draft, Visualization, Validation, Supervision, Software, Methodology, Investigation, Formal analysis, Conceptualization. **Jean-Patrick Pinheiro:** Writing – review & editing, Validation, Methodology, Investigation, Formal analysis, Data curation, Conceptualization. **Lars-André Tokheim:** Writing – review & editing, Validation, Supervision, Resources, Project administration, Methodology, Investigation, Funding acquisition, Conceptualization.

## Declaration of competing interest

The authors declare that they have no known competing financial interests or personal relationships that could have appeared to influence the work reported in this paper.

## Figures and Tables

**Table A1:** Components and model types.

Model type	Components
**Solid model 1**	CaCO_3_, CaO, SiO_2_, Al_2_O_3_, Fe_2_O_3_, Na_2_SO_4_, CaSO_4_
**Solid model 2**	K_2_SO_4_, K_2_O, Na_2_O
**Gas model 1**	CO_2_

**Table A2:** The specific heat equations.

Model type	Specific heat equation [J/mol-K]
**Solid model 1**	$C_{p}=C_{1}+C_{2}T+C_{3}T^{2}+C_{4}T^{3}+C_{5}T^{4}$ $C_{6}\leq T\leq C_{7}$
**Solid model 2**	$C_{p}=C_{1}+C_{2}T+C_{3}T^{2}+\frac{C_{4}}{T}+\frac{C_{5}}{T^{2}}+\frac{C_{6}}{T^{0.5}}$ $C_{7}\leq T\leq C_{8}$
**Gas model 1**	$C_{p}=C_{1}+C_{2}{\left(\frac{C_{3}/T}{\sinh\left(C_{3}/T\right)}\right)}^{2}+C_{4}{\left(\frac{C_{5}/T}{\cosh\left(C_{5}/T\right)}\right)}^{2}$ $C_{6}\leq T\leq C_{7}$

**Table A3:** The enthalpy equations.

Model type	Specific heat equation [kJ/mol]
**Solid model 1**	$H-H_{ref}=C_{1}\left(T-T_{ref}\right)+\frac{C_{2}\left(T^{2}-{T_{ref}}^{2}\right)}{2}+\frac{C_{3}\left(T^{3}-{T_{ref}}^{3}\right)}{3}+\frac{C_{4}\left(T^{4}-{T_{ref}}^{4}\right)}{4}+\frac{C_{5}\left(T^{5}-{T_{ref}}^{5}\right)}{5}$ $C_{6}\leq T\leq C_{7}$
**Solid model 2**	$H-H_{ref}=C_{1}\left(T-T_{ref}\right)+\frac{C_{2}\left(T^{2}-{T_{ref}}^{2}\right)}{2}+\frac{C_{3}\left(T^{3}-{T_{ref}}^{3}\right)}{3}+C_{4}\;\ln\left(\frac{T}{T_{ref}}\right)-C_{5}\left(\frac{1}{T}-\frac{1}{T_{ref}}\right)+\frac{C_{6}\left(T^{0.5}-{T_{ref}}^{0.5}\right)}{0.5}$ $C_{7}\leq T\leq C_{8}$
**Gas model 1**	$H-H_{ref}=C_{1}\left(T-T_{ref}\right)+C_{2}C_{3}\left[\coth\left(\frac{C_{3}}{T}\right)-\coth\left(\frac{C_{3}}{T_{ref}}\right)\right]-C_{4}C_{5}\left[\tanh\left(\frac{C_{5}}{T}\right)-\tanh\left(\frac{C_{5}}{T_{ref}}\right)\right]$ $C_{6}\leq T\leq C_{7}$

**Table A4:** Coefficients in the equations for specific heat/enthalpy. The temperature unit is K.

Comp	$C_{1}$	$C_{2}$	$C_{3}$	$C_{4}$	$C_{5}$	$C_{6}$	$C_{7}$	$C_{8}$
**CaCO**_**3**_	−2.3728	0.4622	−0.000735	5.57E-07	−1.57E-10	298	1200	–
**CaO**	23.0403	0.09213	−0.00010746	5.716E-08	−1.11E-11	298	1900	–
**SiO**_**2**_	−8.469	0.252	−0.000296	1.518E-07	−2.84E-11	298	1900	–
**Al**_**2**_**O**_**3**_	2.495	0.3665	−0.000422	2.208E-07	−4.25E-11	298	1900	–
**Fe**_**2**_**O**_**3**_	51.836	0.153	0.00014	−2.910E-07	1.03E-10	298	1700	–
**MgO**	13.42	0.114	−0.00013	6.94E-08	−1.33E-11	298	1900	–
**Na**_**2**_**SO**_**4**_	53.77	0.3009	−0.00019	2.58E-08	5.28E-12	298	1900	–
**CO**_**2**_	53.7	9.95	1887.73	−41.5	−273.6	50	5000	–
**CaSO**_**4**_	96.2	−0.066	0.000336	−2.55E-07	5.75E-11	298	3000	–
**K**_**2**_**O**	−668.9	0.2336	−4.10E-05	−362877	1.90E+07	28936	298	1900
**Na**_**2**_**O**	1722.67	−0.3185	3.40E-05	937944	−5.64E+07	−70248	298	1900

**Table A5:** Standard enthalpy of the components at 25 °C and 1 bar [37].

Component	Standard enthalpy ($H_{ref}$) [J/mol]	Component	Standard enthalpy ($H_{ref}$) [J/mol]
**CaCO**_**3**_	−1206921	**K**_**2**_**SO**_**4**_	−1437790
**CaO**	−635089	**Na**_**2**_**O**	−417982
**CO**_**2**_	−393505	**K**_**2**_**O**	−361498
**SiO**_**2**_	−910857	**MgO**	−601241
**Al**_**2**_**O**_**3**_	−1675692	**Fe**_**2**_**O**_**3**_	−824248
**CaSO**_**4**_	−1434108		

**Table B1:** Constants to predict CO_2_ emissivity [38].

Gas temperature ($T_{g}$) [K]	$a_{0}$	$a_{1}$	$a_{2}$	$a_{3}$
1000	2.2661	0.1742	−0.039	0.004
1500	2.3954	0.2203	−0.0433	0.00562
2000	2.4104	0.2602	−0.0651	−0.00155

**Fig. B1:**
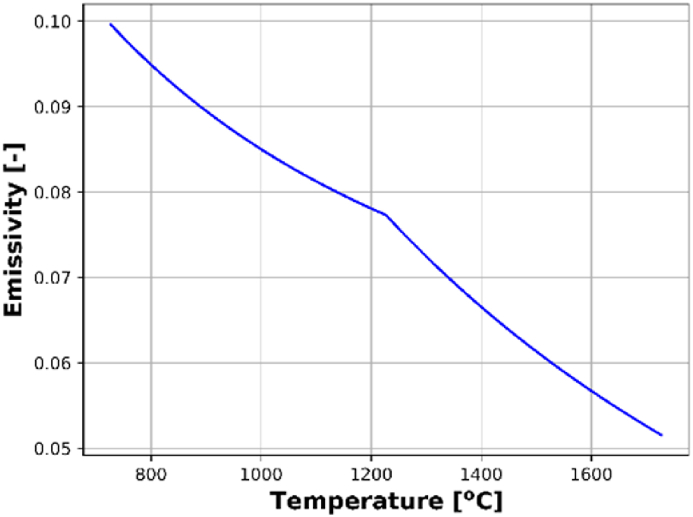
CO_2_ emissivity as a function of gas temperature.

**Fig. B2:**
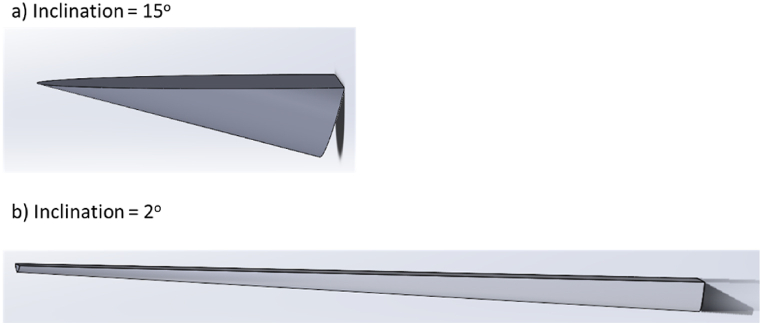
Geometrical view of 200 g of particles inside the calciner inclined at a) 15° and b) 2°.

**Fig. B3:**
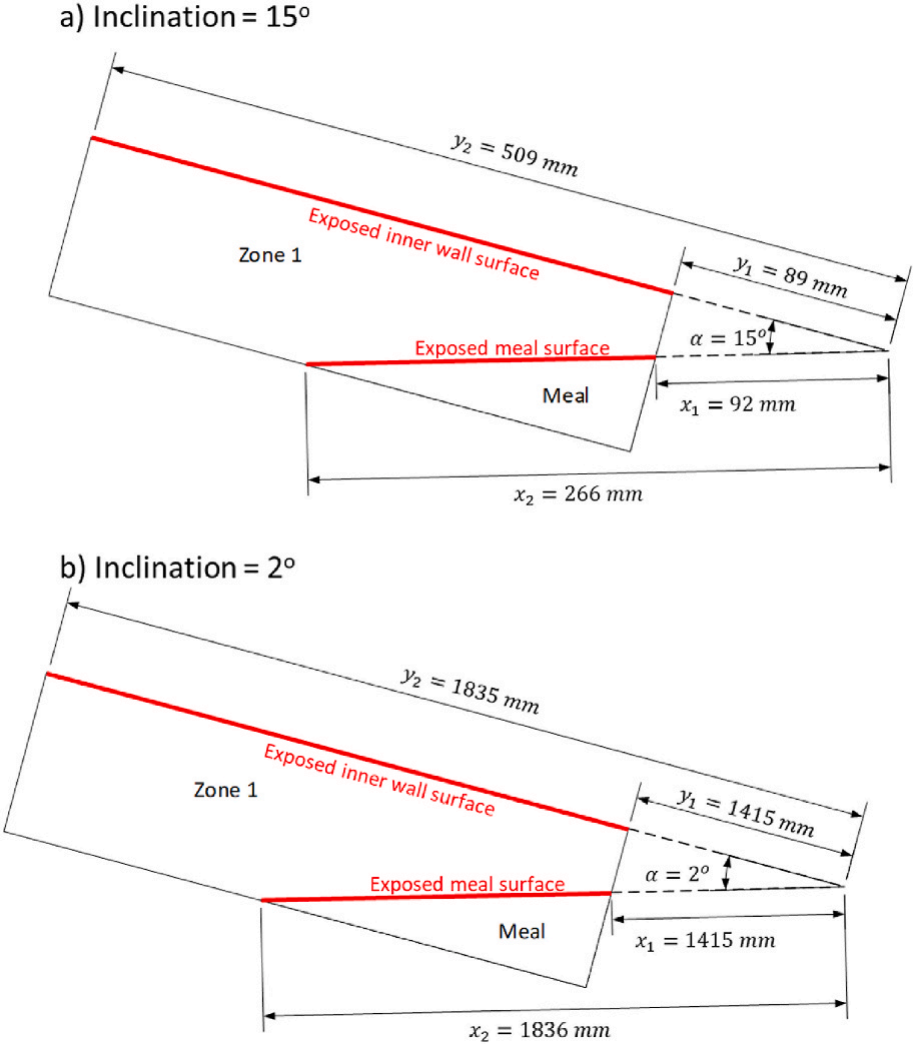
Configuration for radiation view factor calculations, including calculated lengths of the system with 200 g of raw meal inside the calciner inclined at a) 15° and b) 2°.

**Table B2:** Estimated parameters for heat transfer calculations.

Parameters	Units	Inclination = 15°	Inclination = 2°
Particle mass	g	200	200
Area of exposed meal ($A_{em}$)	mm^2^	9961	21455
Area of wall in direct contact with meal ($A_{cw}$)	mm^2^	13547	24610
Area of exposed wall ($A_{ew}$)	mm^2^	77232	66169
Maximum bed height of bed ($h_{p,m}$)	mm	45	19
View factor from meal to exposed wall ($F_{m,w}$)	–	0.91	0.87

## Nomenclature

**Small letters**
$d_{C}$: Internal diameter of scaled-up calciner drum, m
$f_{m}$: Fraction of maximum meal bed height, %
$h_{cw,m}$: Heat transfer coefficient from contact wall to meal, W/(m^2^K)
$h_{PH}$: Heat transfer coefficient during meal preheating, W/(m^2^K)
$h_{C}$: Heat transfer coefficient during meal calcination, W/(m^2^K)
$h_{p,m}$: Maximum bed height in drum, m
$k_{m}$: Thermal conductivity of meal, W/(m·K)
$k_{D}$: Kinetics of calcination reaction, mol/(m^2^·s·Pa)
$l_{C}$: Length of scaled-up calciner drum, m
$m_{Final}$: Final sample mass during calcination degree measurement, g
$m_{Initial}$: Initial sample mass during calcination degree measurement, g
$n_{j}$: Moles of component j, mole
$p_{co2}$: Partial pressure of CO_2_ in the calciner, Pa
$p_{eq}$: Equilibrium pressure for calcite decomposition, Pa
${\dot{q}}_{cw}$: Heat transfer from wall in direct contact with the meal, W
${\dot{q}}_{ew}$: Heat transfer from exposed wall to the meal, W
${\dot{q}}_{net,m}$: Net heat transfer to the meal, W
${\dot{q}}_{net,m,PH}$: Net heat transfer to the meal during preheating stage, W
${\dot{q}}_{net,m,C}$: Net heat transfer to the meal during calcination stage, W
$r_{C}$: Rate of calcination reaction, mol/(m^2^·sec)

**Capital letters**
$A_{c}$: Surface area of the calcite, m^2^
$A_{cw}$: Surface area of the wall in contact with the meal, m^2^
$A_{eff}$: Pore-to-particle area ratio, -
$A_{em}$: Surface area of exposed meal, m^2^
$A_{ew}$: Surface area of exposed wall, m^2^
$A_{w,in}$: Inner surface area of the wall, m^2^
$A_{HT}$: Total heat transfer area for scale-up calculations, m^2^
$E_{b,g}$: Black body emissive power flux from gas, W/m^2^
$E_{b,m}$: Black body emissive power flux from meal, W/m^2^
$E_{b,\text{ew}}$: Black body emissive power flux from exposed wall, W/m^2^
$F_{m,g}$: View factor from meal to gas, -
$F_{m,w}$: View factor from meal to inner wall, -
$F_{w,g}$: View factor from inner wall to gas, -
$H_{j}$: Specific enthalpy of component j, J/mol
$H_{m}$: Meal enthalpy, J
$J_{g}$: Radiosity from gas, W/m^2^
$J_{m}$: Radiosity from meal, W/m^2^
$J_{ew}$: Radiosity from exposed wall, W/m^2^
LOI: Loss on ignition, %
$R_{1}$: Radiation resistance due to emissivity of the exposed wall, 1/m^2^
$R_{2}$: Radiation resistance due to view from exposed wall to gas, 1/m^2^
$R_{3}$: Radiation resistance due to view from meal to exposed wall, 1/m^2^
$R_{4}$: Radiation resistance due to view from meal to gas, 1/m^2^
$R_{5}$: Radiation resistance due to emissivity of the meal, 1/m^2^
$R_{eff}$: Effective radiation resistance from exposed wall to the meal, 1/m^2^
$T_{m}$: Temperature of meal core, K
$T_{m,c}$: Meal calcination temperature, K
$T_{m,in}$: Meal initial temperature, K
$T_{m,s}$: Temperature of meal surface exposed to the exposed inner zone 1 wall, K
$T_{w,in}$: Temperature of inner wall, K
$\Delta T_{LMTD}$: Log mean temperature difference during meal preheating, K
$X_{c}$: Calcination degree, %

**Greek letters**
δ: Conduction thickness, m
$\varepsilon_{w}$: Emissivity of inner wall, -
$\varepsilon_{m}$: Emissivity of meal, -
$\varepsilon_{g}$: Emissivity of gas, -
$\vartheta_{j}$: Stochiometric coefficient in calcination reaction for component j, -
σ: Stefan-Boltzmann's constant, W/(m^2^K^4^)

## Appendix A Species, polynomial coefficients, and thermodynamic data

The components used in the model are summarized in Table A1. Further, the polynomial equations to predict specific heat and enthalpy are shown in Table A2 and Table A3. The polynomial coefficients for specific heat and enthalpy are summarized in Table A4, obtained after fitting all the data to the database published in Barin's handbook [37]. The enthalpy equation is derived by integrating the specific heat equations shown as equation (A1).

(A1) $$H-H_{ref}=\int_{T_{ref}}^{T}C_{P}\left(T\right)dT$$

Here, H is the enthalpy at a given temperature [J/mol], $H_{ref}$ is standard enthalpy [J/mol] (standard condition is defined as 25 °C and 1 bar), and $C_{P}\left(T\right)$ is the polynomial equation for specific heat [J/(mol·K)]. The standard enthalpy is summarized in Table A5, which is taken from Barin's handbook [37].

## Appendix B Calculation of unknowns to determine the heat transfer parameters

The resistance to radiation heat transfer comes either from emissivities ($R_{1}$, $R_{5}$) or view factors ($R_{2}$, $R_{3}$, $R_{4}$). The unknown parameters required to determine the network resistances ($R_{1}\ldots R_{5}$) are:

• CO_2_ emissivity ($\varepsilon_{g}$)
• Inner wall to gas view factor ($F_{w,g}$)
• Meal-to-gas view factor ($F_{m,g}$)
• Area of exposed meal ($A_{\text{em}}$)
• Area of exposed wall ($A_{\text{ew}}$)
• View factor of meal to exposed wall ($F_{m,w}$)

The emissivity of CO_2_ ($\varepsilon_{g}$) is a function of gas temperature ($T_{g}$) [K] and the product of pressure and characteristic length (pL) [m·atm] and is given by equation (B1) [38]. The constants are given in Table B1 [38].

(B1) $$\log\left(\bar{\varepsilon_{g}T_{g}}\right)=a_{0}+a_{1}\;\log\left(pL\right)+a_{2}\;\log^{2}\left(pL\right)+a_{3}\;\log^{3}\left(pL\right)$$

Further, the characteristic length for a cylinder is given by L=0.95D [32], where D is the diameter. Thus, at ambient pressure, the product of the characteristic length and the pressure is 0.065 m atm. At this value, the variation of CO_2_ emissivity is shown in Figure B1. Based on this, the value of the gas emissivity ($\varepsilon_{g}$) was assumed to be at 0.09 as we will operate in the range between 800 and 1000 °C.

Any surface, such as the meal and wall, is fully visible to the gas. So, the view factor from any surface to the gas is assumed to be one (i.e. $F_{w,g}=F_{m,g}=1$).

The geometry of the meal in the inclined cylinder at the experimental conditions is drawn with SolidWorks to estimate geometrical parameters, as shown in Figures B2(a-b).

The view factor for heat transfer from the meal to the inner wall ($F_{m,w}$) is calculated by assuming a configuration of two long plates of unequal width without a common edge [39]. According to this configuration, the view factor ($F_{m,w}$) is given by equation (B2) [39].

(B2) $$F_{m,w}=\frac{\left[\begin{array}{c} {\left({x_{1}}^{2}+{y_{2}}^{2}-2x_{1}y_{2}\;\cos\;\alpha \right)}^{0.5}+{\left({x_{2}}^{2}+{y_{1}}^{2}-2x_{2}y_{1}\;\cos\;\alpha \right)}^{0.5}\\ -{\left({x_{2}}^{2}+{y_{2}}^{2}-2x_{2}y_{2}\;\cos\;\alpha \right)}^{0.5}-{\left({x_{1}}^{2}+{y_{1}}^{2}-2x_{1}y_{1}\;\cos\;\alpha \right)}^{0.5} \end{array}\right]}{2\left(x_{2}-x_{1}\right)}$$

Parameters such as $x_{1}$, $x_{2}$, $y_{1}$, $y_{2}$, and α are estimated based on SolidWorks drawing, shown in Fig. B3(a-b). The resulting parameters for heat transfer calculations are summarized in Table B2.
